# Authentication using c-VEP evoked in a mild-burdened cognitive task

**DOI:** 10.3389/fnhum.2023.1240451

**Published:** 2023-09-07

**Authors:** Zhihua Huang, Zequan Liao, Guojie Ou, Lijun Chen, Ying Zhang

**Affiliations:** ^1^College of Computer and Data Science, Fuzhou University, Fuzhou, China; ^2^School of Humanities and Social Sciences, Fuzhou University, Fuzhou, China; ^3^Department of Physical Education, Fuzhou University, Fuzhou, China

**Keywords:** authentication, c-VEP, c-EEGNet, MBCT, deep learning

## Abstract

In recent years, more and more researchers are devoting themselves to the studies about authentication based on biomarkers. Among a wide variety of biomarkers, code-modulated visual evoked potential (c-VEP) has attracted increasing attention due to its significant role in the field of brain-computer interface. In this study, we designed a mild-burdened cognitive task (MBCT), which can check whether participants focus their attention on the visual stimuli that evoke c-VEP. Furthermore, we investigated the authentication based on the c-VEP evoked in the cognitive task by introducing a deep learning method. Seventeen participants were recruited to take part in the MBCT experiments including two sessions, which were carried out on two different days. The c-VEP signals from the first session were extracted to train the authentication deep models. The c-VEP data of the second session were used to verify the models. It achieved a desirable performance, with the average accuracy and F1 score, respectively, of 0.92 and 0.89. These results show that c-VEP carries individual discriminative characteristics and it is feasible to develop a practical authentication system based on c-VEP.

## 1. Introduction

In this era of information and intelligence, authentication technology is becoming more and more important. The challenges in authentication technology have certainly attracted increasing attention. Traditional authentication techniques such as passwords and tokens often cause substantial losses due to forgetfulness, spoofing, and circumvention (Jain et al., 2016). Authentication systems based on face and fingerprint still face security issues such as cyber attacks, forgery, and stealing (Jain et al., 2004; Kumar, 2020), although they have achieved excellent performance. More reliable authentication technologies are desirable. In recent years, numerous studies have suggested that Electroencephalogram (EEG) carries individual discriminative characteristics (Bidgoly et al., 2020) and confirmed the universality, collectability, and cancellability of EEG biomarker (Gui et al., 2019; Wang et al., 2022a). Authentication based on EEG has emerged as an attractive option of future authentication technologies.

Researchers have shown a keen interest in EEG-based biometric technologies. Maiorana et al. (2015) collected resting-state EEG data from 30 subjects with both eyes-closed and eyes-open in an individual identification system. Relying on a similarity-based algorithm, they achieved an average recognition accuracy of 85.6%. Chen et al. (2016) proposed an EEG-based login system that applied rapid serial visual presentation (RSVP) to acquire EEG data. A method based on shrinkage discriminant analysis was introduced to process 16 subjects' EEG data from this system. The average validation accuracy reached 87.8%. In an EEG-based authentication study, Seha and Hatzinakos (2020) used steady-state auditory evoked potentials from 40 subjects. They adopted canonical correlation analysis to match EEG samples and achieved a reasonable validation accuracy.

The code-modulated visual evoked potential (c-VEP) is an EEG response elicited by visual stimuli following pseudorandom codes (Mart́ınez-Cagigal et al., 2021). The studies in the field of brain-computer interface (BCI) showed that c-VEP can improve the information transfer rate of the online BCI system (Bin et al., 2011). In recent years, the biometric technology based on c-VEP has been explored. In an individual identification study, Zhao et al. (2019) elicited c-VEP of 25 subjects by modulating time-shifted binary pseudorandom sequences in target stimuli and employed an individual-unique template to process c-VEP for individual identification. The recognition result demonstrated that c-VEP provides distinct individual variability. Afterwards, Zhao et al. (2021) quantitatively compared the performance of flash VEP, steady-state VEP, and c-VEP signals in person identification. The comparison showed that c-VEP is significantly superior to other VEP for individual identification.

The existing study (Wang et al., 2022b) revealed that cognitive tasks can complement the brain biometric by further combining “what we think, feel, and respond during cognitive tasks”. Therefore, cognitive tasks have been extensively applied in EEG-based biometric technologies. Min et al. (2017) designed an individual identification system based on steady-state VEP with a cognitive task of recognizing Korean characters. The EEG data from 20 subjects were transformed into feature vectors by causal connectivity analysis for individual identification. They achieved a recognition accuracy of 98.6%. In an EEG-based authentication study, Wu et al. (2018) proposed a combination of RSVP with self-face or nonself-face (face-RSVP). Fifteen subjects participated in the face-RSVP experiment. The EEG signals during the face-RSVP stimuli were used for the authentication study. The average validation accuracy reached 91.31%.

The methods of processing the EEG in authentication involve multiple aspects. Averaging time-locked EEG epochs can elevate the signal noise ratio of event-related potentials (ERP) (Gui et al., 2019). Several studies (Zhao et al., 2019; Seha and Hatzinakos, 2020) showed that averaging EEG epochs can also improve the performance of EEG-based biometric. As a widely studied approach, deep learning has been introduced into the research of EEG-based biometric. Maiorana (2020) adopted a kind of convolutional neural network (CNN) to handle the problem of matching EEG samples in an EEG-based biometric technology. In an EEG-based authentication study, Bidgoly et al. (2022) used a CNN to extract features from motor imagery EEG data of 109 subjects. Lawhern et al. (2018) proposed a specific CNN, EEGNet, for the purpose of processing EEG signals. Several EEG-based authentication studies (Kumar et al., 2021; Seha and Hatzinakos, 2022) have applied EEGNet.

To our knowledge, EEG-based authentication studies suffer from several limitations such as a large number of required electrodes, lengthy authentication time, lack of protocols for evoking c-VEP, and uncertain stability across time. Based on the advantages of c-VEP BCI and deep learning, we hypothesize that a new c-VEP protocol and a special deep learning model can help overcome some limitations of EEG-based authentication. In this study, we proposed a mild cognitive task to evoke c-VEP and designed a new version of EEGNet to process c-VEP for authentication purpose. Our research investigated the performance and stability of the proposed method and the influences of electrode selection and authentication time on its performance.

## 2. Materials and methods

### 2.1. Main framework

**Figure 1:**
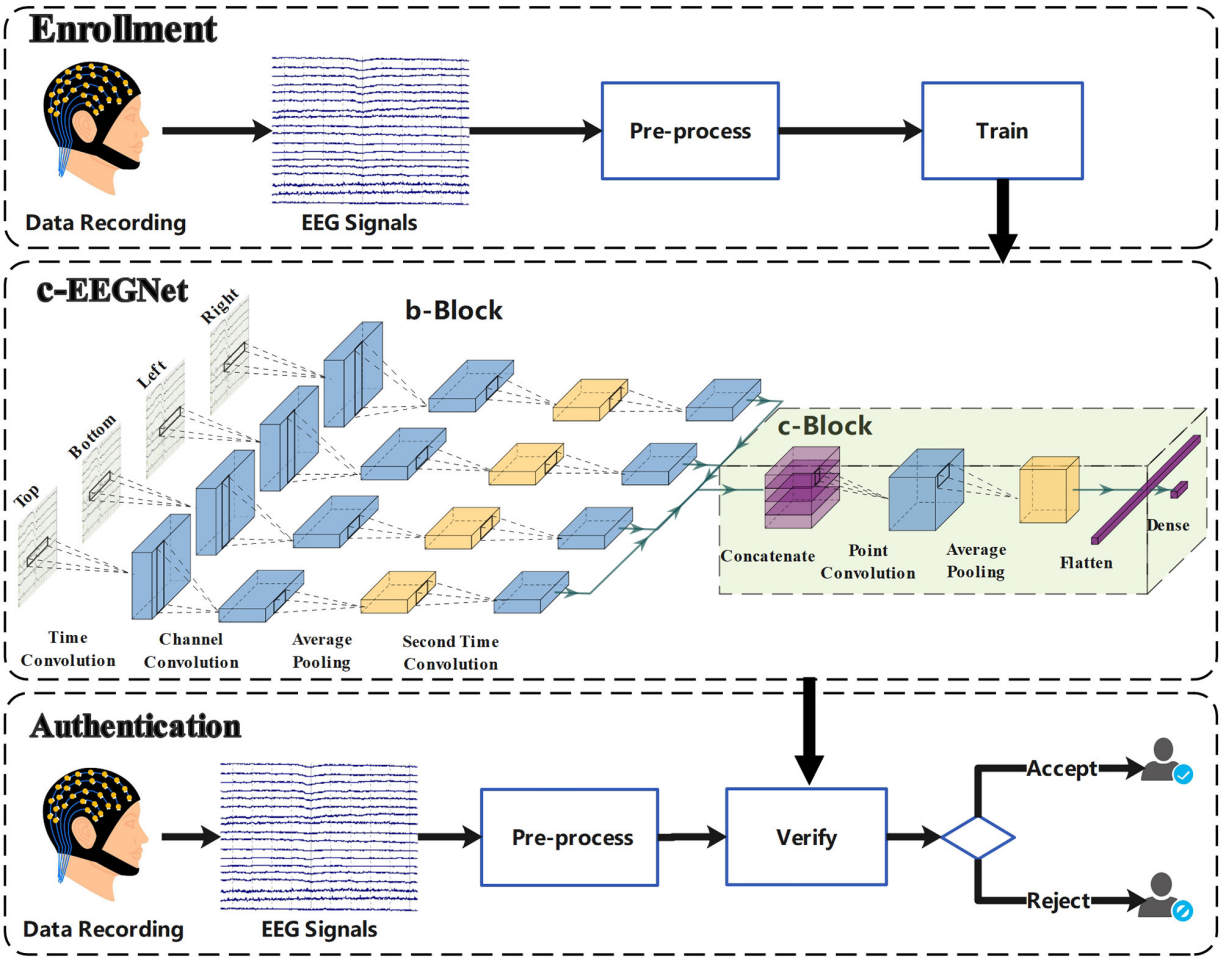
Framework of authentication using c-VEP.

The study on authentication using c-VEP comprises the enrollment and authentication phases. The enrollment phase aims to enroll c-VEP identity models. The purpose of the authentication phase is to verify identities using c-VEP. To simulate the actual authentication scenario, the two phases are always scheduled on different days for each participant. The study framework is shown in Figure 1. In the enrollment phase, we record EEG signals of participants under the condition of code-modulated visual stimuli and train combined EEGNet (c-EEGNet) models for authentication using the EEG signals. In the authentication phase, EEG signals under the same condition are collected again and processed by the c-EEGNet models to verify the identities of the participants.

### 2.2. Experimental protocol

In order to know whether the participants focused their attention on the visual stimuli, we proposed a mild-burdened cognitive task (MBCT). The human visual system is very sensitive to topological distinctions between a ring (with hole) and a disk (without hole) (Chen, 1982). For a normal person, it is very easy to identify the ring from the two shapes. Every time, the MBCT presents a flickering ring and a flickering disk on the screen. The participants are instructed to gaze at the flickering ring and give a response to the position of the ring after the presentation. If the response is right, the visual evoked potentials of the presentation are considered to be reliable. Otherwise, the EEG signals of this presentation would be excluded in the subsequent processing.

**Figure 2:**
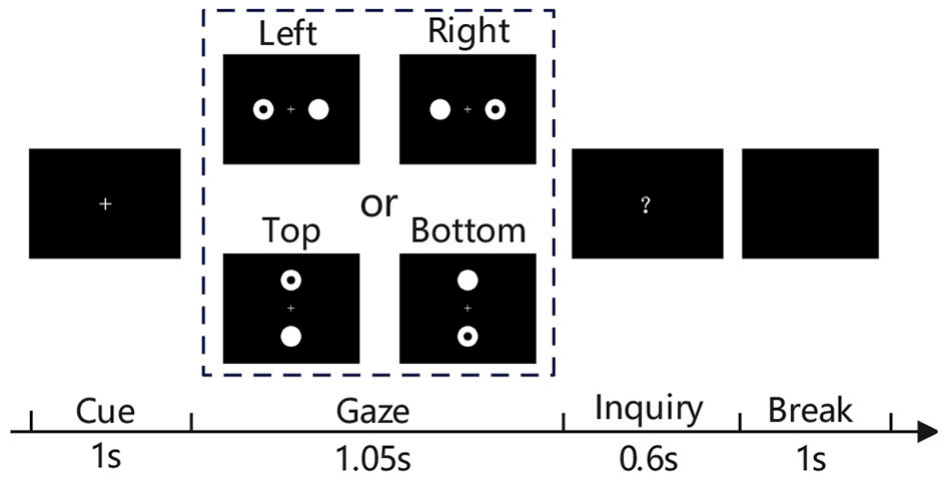
Illustration of the mild-burdened cognitive task experiment.

As illustrated in Figure 2, a MBCT trial consists of four stages. In the cue stage, a fixation cross is presented for 1 s to indicate the start of a new trial. Then, the visual stimuli including a flickering ring and a flickering disk are presented for 1.05 s during the gaze stage. Next, a question mark shown in the screen center prompts participants to click a key to respond to the position of the just presented ring at the inquiry stage, which lasts up to 0.6 s and ends as soon as the participant gives feedback. Finally, the break stage presents a black screen for 1 s.

Especially, the gaze stage presents the visual stimuli in two ways: left vs right (L-R) and top vs bottom (T-B). As shown in Figure 2, the L-R presents the two round objects on the left or right side of the fixation cross, the T-B presents the two round objects on the top or bottom side of the fixation cross. The ring appears randomly on the left or right in the L-R trial, or randomly on the top or bottom in the T-B trial. In a L-R run, the number of left trials is equal to that of right trials. In a T-B run, the number of top trials is also equal to that of bottom trials. Regardless of the presentations, the objects both lie within participants' field of view when they sit at 70 cm in front of the screen. The participants can gaze at the ring without having to turn their heads. The flashes at the left, right, top, or bottom are modulated by four different 63 bits m-sequences. Each bit corresponds to one frame of the flash. “1” means on and “0” represents off. Visual stimuli were presented on the Lenovo LT1913pA monitor with a 60 Hz refresh rate. The flashes of 63 bits last 1.05 s.

### 2.3. Data acquisition

In this study, each run comprised 100 correctly responded trials and each session included four runs, which were separated by short breaks. In each session, the first two runs used the L-R way and the last two runs adopted the T-B way. EEG signals were recorded by a 64-channel neuroscan system while the participants were conducting the MBCT. The electrodes were placed according to the 10-10 standard, with AFz as the ground and the vertex as the reference. The impedances were kept below 10 kΩ. The sampling rate was set to 1,000 Hz. A 50 Hz notch filter was used to eliminate power-line noise.

Seventeen participants (six females) were recruited to take part in the MBCT experiment. Their ages ranged from 20 to 25 years, with the mean of 23. Visual or neurological disorders, head trauma, and any drug use that would affect nervous system function were excluded. In accordance with the Helsinki Declaration of Human Rights, informed consent was obtained from each participant. Each participant performed two sessions on different days separated by intervals ranging from 1 to 337 days.

### 2.4. Pre-processing

Raw EEG signals were pre-processed as follows. Firstly, a band-pass filter of 5–86 Hz was applied to the raw EEG signals (64 channels). Secondly, independent component analysis (ICA) was used to remove the ocular and muscular artifacts. Thirdly, the EEG signals from the nine electrodes over the parietal and occipital areas (Pz, PO5, PO3, POz, PO4, PO6, O1, Oz, and O2) were extracted into EEG epochs. Considering the delay of the visual pathway (Di Russo and Spinelli, 1999), the epochs were defined as the EEG signals from 0.14 to 1.19 s (the stimuli lasted 1.05 s), where 0 referred to the stimulus onset. The epochs in which EEG amplitudes exceeded a threshold of ±100 *μ*V were excluded. Fourthly, the baseline correction and downsampling were conducted respectively by subtracting the mean of the channel and averaging every four sampling points. Finally, every epoch was annotated with left, right, top, or bottom according to the position of the ring and labelled with the identities of the participants. The epochs from the first session and the second session were added into the training set and test set, respectively.

### 2.5. Authentication model

Inspired by the work in Lawhern et al. (2018), we designed a special neural network, called c-EEGNet, to learn the authentication models from the c-VEP signals of participants. The c-EEGNet, shown in Figure 1, consists of branch block (b-Block) and combined block (c-Block).

The b-Block includes four structurally identical branches, which respectively receive the left, right, top, and bottom epochs. Every branch is formed by sequentially connecting time convolution layer, channel convolution layer, average pooling layer, and second time convolution layer. Here, we refer to the intermediate results of the layers as virtual epoch or channel. The time convolution layer provides *N*_1_ time convolution kernels, each of which applies convolution operation in time dimension and produces a virtual epoch. The channel convolution layer has *N*_2_ channel convolution kernels for each virtual epoch. Each channel convolution kernel transforms the corresponding virtual epoch to a virtual channel by a linear combination. The channel convolution layer further processes all virtual channels by exponential linear unit (ELU). The average pooling layer reduces the length of the virtual channels by averaging every *N*_3_ data points. The second time convolution layer applies convolution operation in time dimension again, with a kernel for a virtual channel. The successive operations of the four layers transform the input epoch of the branch to a horizontal data slice.

The c-Block comprises concatenate layer, point convolution layer, average pooling layer, flatten layer, and dense layer. The concatenate layer piles the four horizontal data slices to a data cube. The point convolution layer provides *N*_4_ point convolution kernels. Each point convolution kernel transforms the data cube to a vertical data slice by combining each of the four horizontal data slices to a virtual channel. Furthermore, the point convolution layer applies ELU operation to all points of the data cube. The average pooling layer reduces the size of the data cube by averaging every *N*_5_ data points in time dimension. The flatten layer unrolls the data cube into a vector. The dense layer transforms the vector to a binary classification result using a softmax function. Additionally, it should be pointed out that each convolutional layer is followed by a batch normalization layer, which is conducive to network convergence.

To build authentication models, we extracted a training data packet for each participant from the training set. A sample was composed of a left epoch, a right epoch, a top epoch, and a bottom epoch from a participant. When all numbers of left, right, top, and bottom epochs are 100, the number of the composed samples is 10^8^ for a participant. In order to understand the effect of signal-to-noise ratio (SNR), the epochs used to establish the samples were randomly obtained by averaging (*N* = 1, ⋯ , 20) original epochs of the same class (left, right, top, and bottom) in a sliding window with the length of *N* and the sliding step of 1. For a participant, his/her samples were labelled as positive samples and those of other participants were labelled as negative samples. A training data packet contained 1,600 randomly-selected positive samples and negative ones, where 1,600 negative samples were from the other 16 participants, 100 samples per one. Each training data packet was used to train a c-EEGNet model. During the actual calculation, we set *N*_1_ = 32, *N*_2_ = 2, *N*_3_ = 4, *N*_4_ = 64, *N*_5_ = 8, the sizes of the time convolution kernels and the second time convolution kernels were respectively 1 × 60 and 1 × 16, the dropout rate after the average pooling layers was 0.5. After the model construction, the samples extracted in the same way from the test set were used to verify our approach.

## 3. Results

To evaluate the proposed approach, we conducted the test of c-VEP authentication on the EEG dataset of 17 participants using c-EEGNet. For comparison, the same examination was also carried out with task-related component analysis (TRCA) (Zhao et al., 2019). TRCA builds a set of spatial filters by solving the inter-trial covariance maximization problem. Zhao et al. (2019) constructed a template-matching framework using the spatial filters to serve individual identification based on c-VEP. Their work shows that TRCA is well suited for extracting c-VEP individual traits. Given the similarity between identification and authentication, we choose TRCA as the object of comparison with c-EEGNet.

**Figure 3:**
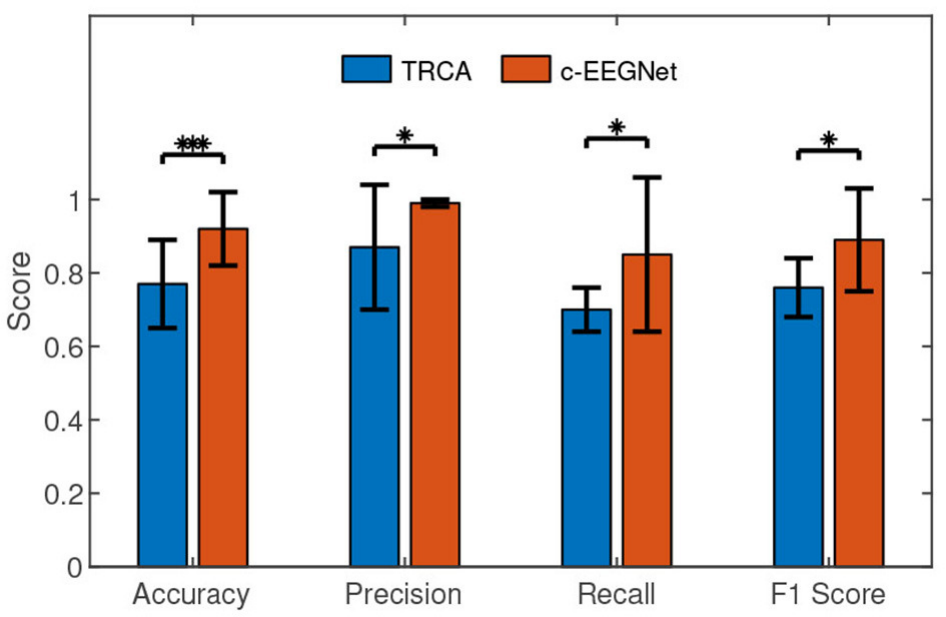
The performance comparisons between c-EEGNet and TRCA. The histograms represent the means and the error bars indicate the standard deviations. We used Wilcoxon signed-rank test in the comparisons. “*” and “***” mean *p* <0.05 and *p* <0.001, respectively.

We calculated accuracy, precision, recall, and F1 score (Fawcett, 2006) for testing results. In Table 1, each row exhibits the accuracies, precisions, recalls, and F1 scores of the two methods for one participant. Figure 3 demonstrates that the accuracy, precision, recall, and F1 scores of c-EEGNet (with the means of 0.92, 0.99, 0.85, and 0.89 respectively) are significantly higher than those of TRCA (with the means of 0.77, 0.87, 0.7, and 0.76 respectively). The Wilcoxon signed rank test results in accuracy (Z value: –3.27, *p* value: 0.001), precision (Z value: –1.98, *p* value: 0.04), recall (Z value: –2.25, *p* value: 0.02), and F1 (Z value: –2.58, *p* value: 0.01) also reveal the significant advantage of c-EEGNet over TRCA.

**Table 1:** The c-VEP authentication performance of TRCA and c-EEGNet on 17 participants when the number of averaged original epochs is 20.

**Participant**	**TRCA**				**c-EEGNet**			
		**Accuracy**	**Precision**	**Recall**	**F1**	**Accuracy**	**Precision**	**Recall**	**F1**
1	0.8	1	0.6	0.75	0.98	1	0.97	0.98
2	0.81	1	0.62	0.77	0.98	1	0.97	0.98
3	0.85	1	0.71	0.83	0.97	1	0.94	0.97
4	0.81	1	0.62	0.76	0.99	0.99	0.98	0.99
5	0.88	0.99	0.77	0.87	0.99	0.99	1	0.99
6	0.44	0.46	0.75	0.57	0.93	1	0.86	0.92
7	0.88	1	0.76	0.86	0.99	0.99	0.99	0.99
8	0.76	0.77	0.76	0.76	0.9	1	0.8	0.89
9	0.83	1	0.67	0.80	0.75	0.99	0.51	0.67
10	0.58	0.57	0.66	0.61	0.74	0.97	0.49	0.65
11	0.9	0.98	0.81	0.89	0.99	0.99	1	0.99
12	0.71	0.72	0.70	0.71	0.78	1	0.57	0.73
13	0.83	1	0.66	0.79	0.99	0.99	1	0.99
14	0.61	0.6	0.67	0.63	0.96	0.93	1	0.96
15	0.88	0.98	0.78	0.87	0.99	0.99	0.99	0.99
16	0.78	0.8	0.76	0.78	0.67	1	0.34	0.51
17	0.83	1	0.66	0.79	0.99	1	0.98	0.98

**Figure 4:**
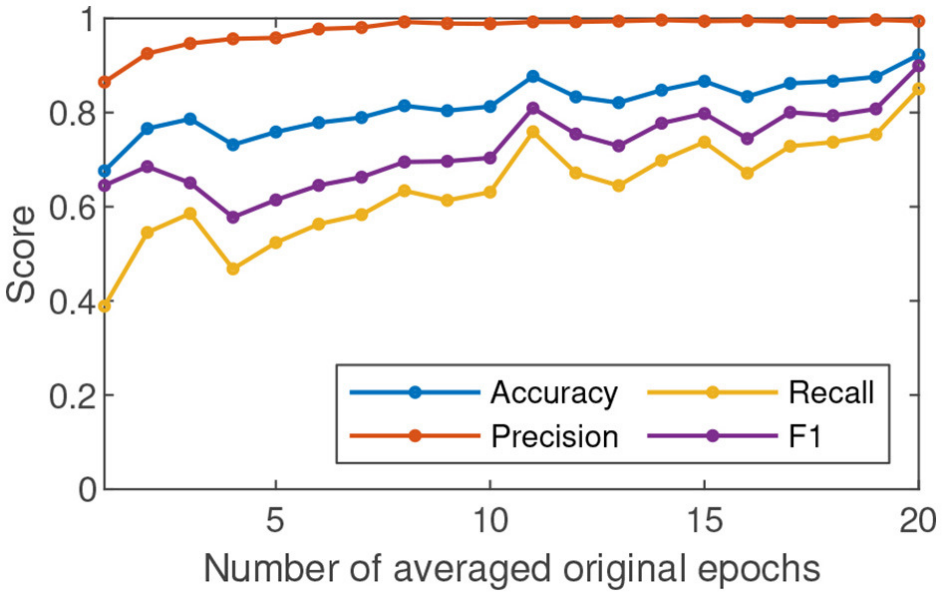
The average accuracy, precision, recall, and F1 score of 17 participants in c-VEP authentication change over the increasing number of averaged original epochs.

Averaging several original epochs can enhance the SNR of c-VEP (Gui et al., 2019). To understand the effect of SNR on c-VEP authentication, we performed the c-VEP authentication experiment in the cases where *N* original epochs of the same class were averaged (*N* = 1, ⋯ , 20). Figure 4 shows the changes of the average accuracy, precision, recall, and F1 score of 17 participants over the increasing number of averaged original epochs. In the case *N* = 1, the accuracy, precision, recall, and F1 score are 0.67, 0.86, 0.38, and 0.64, respectively. In the view of general trend, all the four metrics are improving as the number of averaged original epochs increases. The precision reaches the level of 0.95 in the case *N* = 4 and keeps the level of 0.99 in the cases *N* ≥ 11. The accuracy, recall, and F1 score are 0.73, 0.46, and 0.57 in the case *N* = 4 after a fluctuation, then maintain a growing trend and reach the level of 0.87, 0.75, and 0.8 in the case *N* = 11, next show slow growth amidst minor shocks, and are 0.92, 0.85, and 0.89 in the case *N* = 20. The result demonstrates that the case *N* = 20 results in higher accuracy, recall, and F1 scores than the case *N* = 11. However, no significant difference exists between the two cases in terms of precision only. The precision means of the two cases both are 0.99. In the case *N* = 11, there exist eight participants whose precisions are 1. In the case *N* = 20, there are also eight participants whose precisions are 1.

In order to explore the influence of different brain areas on c-VEP authentication, we selected frontal, parietal, and occipital areas according to the previous work (Huang et al., 2020) and performed the c-VEP authentication experiment in the case *N* = 20 respectively on the frontal (the F1, Fz, and F2 channels), parietal (the P1, Pz, and P2 channels), and occipital (the O1, Oz, and O2 channels) areas. For each brain area, only the signals from the three channels were retained in the EEG epochs during pre-processing. The other operations of the c-VEP authentication experiment are exactly the same. Table 2 exhibits the c-VEP authentication results of the three brain areas. In Table 2, the column headers F, P, and O respectively represent the frontal, parietal, and occipital areas, each row lists the accuracies, precisions, recalls, F1 scores of one participant on the frontal, parietal, and occipital areas. The largest values of the three brain areas are presented in bold font. In Table 2, most bold fonts lie in the O columns and the means of accuracy, precision, recall, and F1 score of the occipital area are higher than the corresponding ones of other brain areas.

**Table 2:** The c-VEP authentication performance of different brain areas when the number of averaged original epochs is 20.

**Participant**	**Accuracy**			**Precision**			**Recall**			**F1**		
		**F**	**P**	**O**	**F**	**P**	**O**	**F**	**P**	**O**	**F**	**P**	**O**
1	0.52	0.53	**0.97**	0.86	0.99	**1**	0.05	0.06	**0.95**	0.1	0.11	**0.97**
2	0.88	**0.98**	0.57	0.99	0.98	**1**	0.78	**0.98**	0.14	0.87	**0.98**	0.25
3	0.66	**1**	0.99	**1**	**1**	**1**	0.32	**1**	0.99	0.48	**1**	0.99
4	0.81	0.97	**0.99**	0.87	0.99	**1**	0.74	0.96	**0.99**	0.8	0.97	**0.99**
5	**1**	0.59	**1**	**1**	0.95	**1**	**1**	0.2	**1**	**1**	0.33	**1**
6	0.54	0.5	**0.98**	0.79	0.62	**1**	0.1	0.04	**0.96**	0.19	0.09	**0.98**
7	0.67	0.63	**0.98**	0.98	**1**	0.98	0.36	0.26	**0.98**	0.52	0.41	**0.98**
8	0.91	0.87	**0.96**	0.97	0.94	**0.98**	0.84	0.79	**0.94**	0.9	0.86	**0.96**
9	0.5	**0.79**	0.76	0.62	**0.99**	**0.99**	0.04	**0.6**	0.53	0.09	**0.75**	0.69
10	**0.75**	0.55	0.56	**0.99**	0.97	0.71	**0.52**	0.12	0.22	**0.68**	0.21	0.34
11	0.57	0.98	**0.99**	0.92	0.98	**1**	0.15	**0.99**	**0.99**	0.26	0.98	**0.99**
12	**0.66**	0.56	0.58	0.98	0.93	**1**	**0.34**	0.13	0.16	**0.5**	0.23	0.28
13	0.52	**0.99**	0.94	0.82	0.99	**1**	0.07	**1**	0.88	0.13	**0.99**	0.93
14	0.5	0.72	**0.97**	0.7	0.9	**0.96**	0.02	0.51	**0.98**	0.04	0.65	**0.97**
15	0.91	0.96	**0.99**	0.99	0.99	**1**	0.83	0.94	**0.99**	0.9	0.96	**0.99**
16	0.68	**0.72**	0.52	0.95	**1**	0.82	0.38	**0.44**	0.07	0.54	**0.61**	0.13
17	0.98	**0.99**	0.98	0.98	**0.99**	0.97	0.99	**1**	**1**	0.98	**0.99**	0.98

The largest values of the three brain areas are presented in bold font.

## 4. Discussion

For authentication, precision is a very important performance indicator (Hernández-Álvarez et al., 2022). In the view of precision, the authentication using c-VEP evoked in the MBCT performed very well. Under the condition of extremely low SNR (the case *N* = 1), the average precision is 0.86. A small improvement in SNR leads to a significant increase in precision. The average precision can keep the level of 0.99 when the SNRs are good enough (the cases *N* ≥ 11). The high precisions show that the proposed approach is expected to serve as an authentication of critical and confidential operations. MBCT can ensure that the samples in the training packets all correspond to a mental state in which the participants focus their attention on the visual stimuli. We think that this property of the training packets could account for the high precisions. If the cognitive task was removed from the paradigm, the training packets would hardly avoid containing noisy samples, leading to a decrease in precision.

Accuracy, recall, and F1 score reveal the performance of the c-VEP authentication from different viewpoints. According to Figure 4, the three indicators also show a growing trend as the SNR gradually increases. In Figure 4, the curves of accuracy, recall, and F1 score present a peak in the case *N* = 11 and another peak in the case *N* = 20. F1 score is the harmonic average of precision and recall. The ideal goal is to get a high F1 score for each participant. Unfortunately, the F1 scores of the participants 9, 10, 12, and 16 are not high enough. An analysis revealed that the EEG signal quality of the participants is significantly lower than that of others. The reasons for the difference could be the change in mental state of the same subject and the session-to-session electrode shift during acquiring EEG (Gembler et al., 2020). We will investigate the causes of this variability in the future. From another perspective, the participants have very high precisions although their F1 scores are not good enough. That is, other participants are largely not mistaken for them although they are sometimes rejected. If someone is rejected, the new operation can finally get him accepted. The results of the participants 9, 10, 12, and 16 are not ideal but acceptable.

In the pre-processing of this study, we used an automated ICA computer program to remove ocular and muscular artifacts, which does not require an expert to analyze the ICA components. The purpose of using the ICA algorithm in our study is to make full use of the collected EEG data. In a real-life scenario, we can even dispense with the ICA algorithm. A simple solution is to directly discard the EEG data containing artifacts and collect it again in the authentication phase.

**Figure 5:**
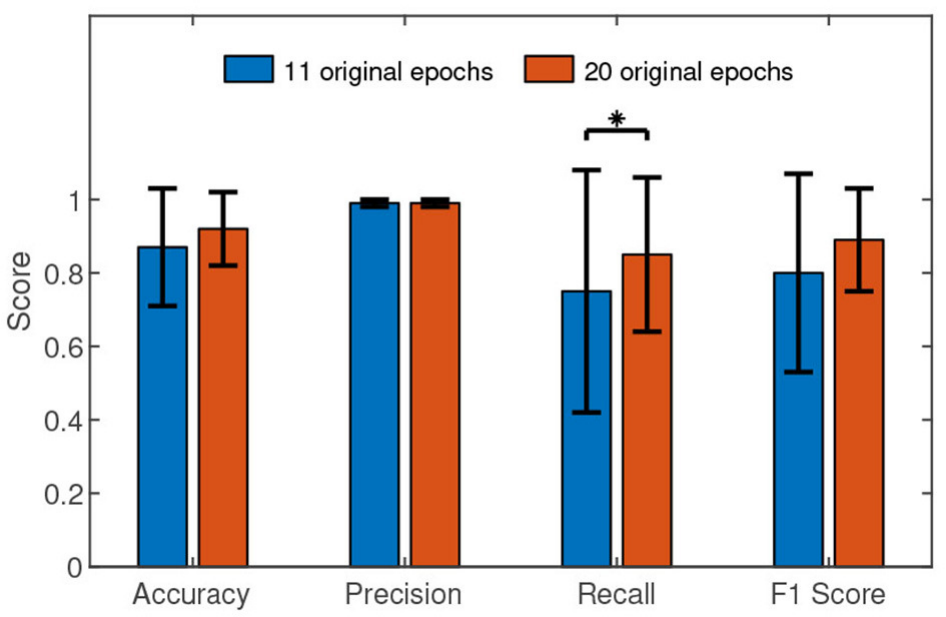
The performance comparisons between the case *N* = 11 and the case *N* = 20. The histograms represent the means and the error bars indicate the standard deviations. We used Wilcoxon signed-rank test in the comparisons. “*” means *p* <0.05.

The performance comparisons between the two cases are presented in Figure 5. Only in recall, the Wilcoxon signed rank test (Z value: –0.76, *p* value: 0.04) revealed the slight advantage of the case *N* = 20 over the case *N* = 11. The Wilcoxon signed rank test results in accuracy (Z value: –1.94, *p* value: 0.05), precision (Z value: –0.30, *p* value: 0.76), and F1 (Z value: –1.83, *p* value: 0.06) between the two cases found no significant difference. Especially, there is almost no precision difference between the two cases.

High feasibility means achieving high recognition performance at a low time cost in the authentication phase. High recognition performance needs high c-VEP SNR. Therefore, it is essential to average more original epochs for high recognition performance. On the other hand, the larger the number of averaged original epochs, the greater the time cost. We have to compromise between time cost and recognition performance. The results show that *N* = 11 is a trade-off between the two.

In general, the proposed c-VEP authentication achieved a desirable performance. Averaging epochs can enhance the SNR of c-VEP and therefore improve the performance of c-VEP authentication. Our results are consistent with related studies (Zhao et al., 2019; Seha and Hatzinakos, 2020). When the number of averaged original epochs is 11, the performance of the approach has been quite good. When the number of averaged original epochs continues to increase, the trend of performance improvement is still maintained but slower.

**Figure 6:**
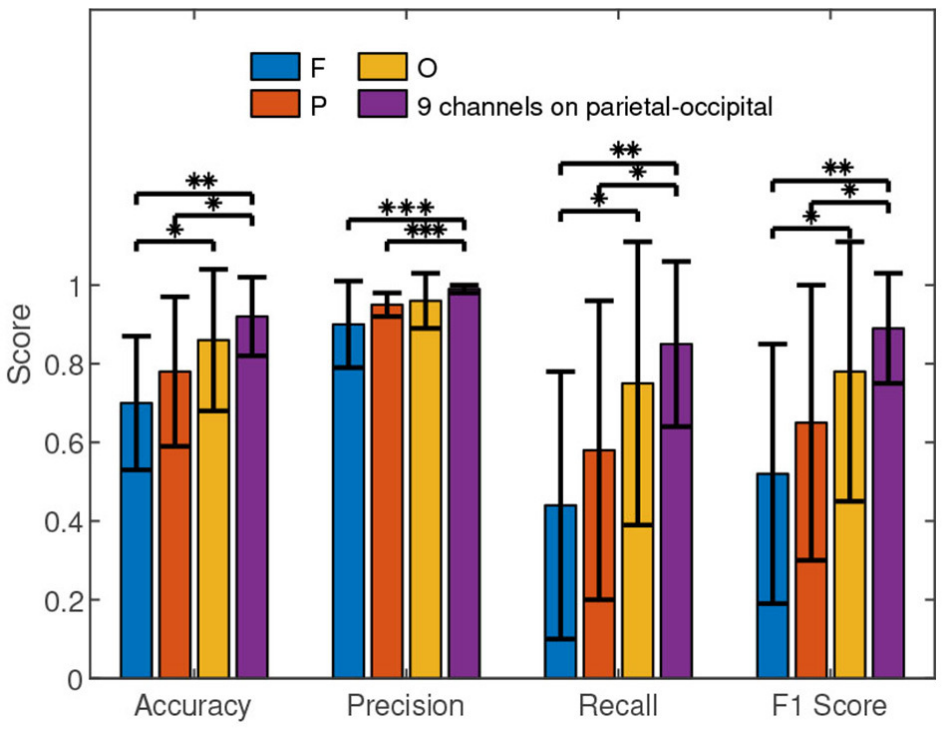
The performance comparisons of different brain areas. F, P, O, and PO respectively represent the frontal (F1, Fz, and F2), parietal (P1, Pz, and P2), occipital (O1, Oz, and O2), and parietal-occipital (Pz, PO5, PO3, POz, PO4, PO6, O1, Oz, and O2) areas. The histograms represent the means and the error bars indicate the standard deviations. We used Wilcoxon signed-rank test in the comparisons. “*”, “**” and “***” mean *p* <0.05, *p* <0.01, and *p* <0.001, respectively.

In order to understand the results shown in Table 2 in depth, we carried out further performance comparisons, which are presented in Figure 6. The average performance values of the occipital area are higher than those of the frontal and parietal areas. We conducted the Wilcoxon signed-rank test in the statistical comparisons. The results between O and F in accuracy (Z value: –2.21, *p* value: 0.02), recall (Z value: –2.12, *p* value: 0.03), and F1 score (Z value: –1.98, *p* value: 0.04) reveal that the occipital area performed significantly better than the frontal area for the authentication. However, no significant performance difference between occipital area and parietal area was discovered by Wilcoxon signed-rank test.

Figure 6 shows that the average performance values of the parietal-occipital area are higher than the counterparts of the frontal, parietal, and occipital areas. Furthermore, the Wilcoxon signed-rank test results in accuracy (Z value: –3.14, *p* value: 0.002), precision (Z value: –3.25, *p* value: 0.001), recall (Z value: –2.99, *p* value: 0.003), and F1 score (Z value: –3.05, *p* value: 0.002) reveal that a significant performance difference indeed exists between the parietal-occipital and frontal areas for the authentication. Similarly, the Wilcoxon signed-rank test results in accuracy (Z value: –2.48, *p* value: 0.01), precision (Z value: –3.35, *p* value: 0.001), recall (Z value: –2.07, *p* value: 0.04), and F1 score (Z value: –2.39, *p* value: 0.01) show the significant advantage of the parietal-occipital area over the parietal area in the authentication. However, the Wilcoxon signed-rank test results in accuracy (Z value: –1.04, *p* value: 0.29), precision (Z value: –0.63, *p* value: 0.52), recall (Z value: –1.08, *p* value: 0.27), and F1 score (Z value: –0.91, *p* value: 0.36) show that the performance difference between the parietal-occipital and occipital areas is not statistically significant for the authentication.

In short, the signals from occipital area result in better performance of c-VEP authentication than those from the frontal area and parietal area. Although the 9 channels on parietal-occipital area can achieve slightly higher accuracies, precisions, recalls, and F1 scores than the 3 channels on occipital area, the differences are not statistically significant. The fact will be an important reference when we have to look for a balance between performance and cost for c-VEP authentication.

Our work shows that the authentication based on c-VEP is an attractive option of future authentication technologies. However, there is still a long way before it is practical. This study indicates that practical recognition performance can be achieved by averaging 11 EEG epochs. This means that the EEG acquisition time of at least 46.2 s is required for practical authentication. In similar studies, Maiorana et al. (2015) used 40 s EEG signals from 19 channels, Seha and Hatzinakos (2020) used 30 s EEG signals from 7 channels, and Zhao et al. (2019) used 10.5 s EEG signals from 9 channels. Although our study revealed the feasibility of using only the three occipital channels, the acquisition time is still too long for practical applications. Therefore, it is necessary in the future to develop new approaches to reduce EEG acquisition time without sacrificing recognition performance. In addition, the current EEG signals are acquired with wet electrodes. The inconvenient operation of wet electrodes will limit the application of authentication based on c-VEP. A proven method must be migrated to the condition of dry electrodes before practical use. As you can imagine, there will be many challenges in the process.

## 5. Conclusion

This study proposed a mild-burdened cognitive task to ensure that the obtained c-VEP signals are the participants' EEG signals when they were paying attention to the visual stimuli. The effectiveness of c-VEP can prevent authentication from being misled. Furthermore, this study designed a deep artificial neural network, c-EEGNet, on the basis of EEGNet (Lawhern et al., 2018). The c-EEGNet was used to learn authentication models from c-VEP signals. The learned models were applied to c-VEP authentication. The experiments show that the proposed approach could achieve a desirable performance and enable real-world authentication systems. Additionally, this study also explored the influence of SNR and brain areas on the performance of c-VEP authentication. These findings serve as a starting point for our future work and are also a reference for peers.

## Data availability statement

The raw data supporting the conclusions of this article will be made available by the authors, without undue reservation.

## Ethics statement

The studies involving humans were approved by Institutional Review Board at Fuzhou University and Helsinki Declaration of Human Rights. The studies were conducted in accordance with the local legislation and institutional requirements. The participants provided their written informed consent to participate in this study.

## Author contributions

ZH, LC, and YZ conceived and designed the research. ZH, ZL, and GO conducted the research. ZL and GO jointly undertook the data collection, programming, and statistical analysis. ZL penned an initial draft of the manuscript. ZH finished the manuscript in its submitted state. ZH and YZ critically reviewed and edited the manuscript. YZ provided essential resources. All authors took part in the manuscript revision.
